# Factors predicting failure of internal fixations of fractures of the lower limbs: a prospective cohort study

**DOI:** 10.1186/s12891-021-04688-6

**Published:** 2021-09-16

**Authors:** Barbara Prediger, Thorsten Tjardes, Christian Probst, Anahieta Heu-Parvaresch, Angelina Glatt, Dominique Rodil dos Anjos, Bertil Bouillon, Tim Mathes

**Affiliations:** 1 Institute for Research in Operative Medicine, Witten/Herdecke University, Ostmerheimer Str. 200, Building 38, NRW 51109 Cologne, Germany; 2 Cologne-Merheim Clinic, Kliniken der Stadt Köln gGmbH, Cologne, Germany; 3 Hospital Gummersbach, Klinikum Oberberg GmbH, Cologne, Germany

**Keywords:** Prediction factors, Failure, Osteosynthesis, Lower extremities, Fractures

## Abstract

**Background:**

We assessed predictive factors of patients with fractures of the lower extremities caused by trauma. We examined which factors are associated with an increased risk of failure. Furthermore, the predictive factors were set into context with other long-term outcomes, concrete pain and physical functioning.

**Methods:**

We performed a prospective cohort study at a single level I trauma center. We enrolled patients with traumatic fractures of the lower extremities treated with internal fixation from April 2017 to July 2018. We evaluated the following predictive factors: age, gender, diabetes, smoking status, obesity, open fractures and peripheral arterial diseases. The primary outcome was time to failure (nonunion, implant failure or reposition). Secondary outcomes were pain and physical functioning measured 6 months after initial surgery. For the analysis of the primary outcome, we used a stratified (according fracture location) Cox proportional hazard regression model.

**Results:**

We included 204 patients. Overall, we observed failure in 33 patients (16.2 %). Most of the failures occurred within the first 3 months. Obesity and open fractures were associated with an increased risk of failure and decreased physical functioning. None of the predictors showed an association with pain. Age, female gender and smoking of more than ≥ 10 package years increased failure risk numerically but statistical uncertainty was high.

**Conclusions:**

We found that obesity and open fractures were strongly associated with an increased risk of failure. These predictors seem promising candidates to be included in a risk prediction model and can be considered as a good start for clinical decision making across different types of fractures at the lower limbs. However, large heterogeneity regarding the other analyzed predictors suggests that “simple” models might not be adequate for a precise personalized risk estimation and that computer-based models incorporating a variety of detailed information (e.g. pattern of injury, x-ray and clinical data) and their interrelation may be required to significantly increase prediction precision.

**Trial registration:**

NCT03091114.

## Background

Osteosynthesis is the fixation of fractures or osteotomies by mechanical devices and usually describes the internal fixation of bone segments.

The aim of surgical fracture care with osetosynthetic devices is to restore the anatomic integrity of the injured bone, thereby allowing for early motion or weight bearing with the aim to enable early training and rehabilitation of the injured limb or joint. Surgical fracture care fails with a rate of 10-19 % [1–4]. Failure of an osteosynthesis usually results in prolonged treatment, revision surgery, worse functional outcome and pain [1–4].

Generally, any osteosynthesis is a trade off on a continuum between mechanical stability of the construct, residual motion of the fracture fragments, which is necessary to stimulate bone healing and the extend of soft tissue damage that has to be accepted to place the implants.

Any osteosynthesis failure is at least in part causally related to pre, intra- and postoperative decisions taken by the surgeon. The surgeon has to balance the individual patient prognostic and predictive factors against the mechanical necessities of bone healing physiology and his or her personal skill set to actually implement the intended osteosynthesis in the open reduction.

Given the complexity of the process of fracture healing and the broad variability of fracture patterns there is usually not sufficient explicit knowledge to provide a good basis for predicting fracture healing pre-surgery. Since this is not the case, many general parameters such as age or smoking status are considered as a starting point for the surgeon to decide on the optimal therapeutic strategy. Most of these parameters have in common that they refer primarily not to bone physiology related conditions. Pathomechanically, these parameters are usually not clearly traceable to the physiology of fracture healing. This gap of causality necessarily introduces a high degree of uncertainty for any clinical decision. Nevertheless, several studies have identified factors that are associated with osteosynthesis failure in lower extremity fractures [5–10].

All these studies were retrospective or based on routinely collected data.

The objective of this prospective cohort study was to analyze predictive factors for treatment failure in patients who have received an osteosynthesis at the lower extremity. Given the limited resources available to gather relevant information in practice, the present study focuses on predictors, which are easily to collect in practice and thus offer the opportunity to make a “simple” individual risk estimation prior to surgery. In addition, the study should prove if it is sensible to develop a “simple” risk prediction model.

In addition, we analyzed the association between several predictive factors long-term pain and physical functioning to set the results into a broader morbidity perspective.

## Methods

### Study design

We performed a prospective cohort study. All patients fulfilling the inclusion criteria were consecutively included. Prior to enrollment, we registered the study (registry no. : NCT03091114) and received the approval by the ethic committee of Witten/Herdecke University. The study is reported in accordance with the Strengthening the Reporting of Observational studies in Epidemiology (STROBE) statement.

### Patients and setting

#### Setting

We screened all patients who underwent surgical fracture care of the lower extremity in a level I trauma center in urban Germany between April 2017 and July 2018 for eligibility.

#### Eligibility criteria

Patients had to fulfill all of the following inclusion criteria.

- Age ≥ 18 years.
- Internal fixation of an isolated traumatic tibial, femoral, calcaneal, malleolar or fibular fracture.
- Sufficient German language skills and cognitive abilities to participate in follow up.

Exclusion criteria:

- Periprosthetic fractures.
- Fractures in context of a polytrauma.

We defined no further exclusion criteria to avoid reducing generalizability.

### Predictive factors

A prognostic factor can be defined as a measure that is associated with a subsequent endpoint among (untreated) people with a given start point (here osteosynthesis of lower extremities) [11]. A predictive factor is a subtype of a prognostic factor that predicts an outcome in treated patients, here failure of the internal fixation.

We chose potentially relevant predictive factors based on a literature review and an expert discussion [5–10]. We chose predictive factors for which we anticipated applicability across different types of lower limb fractures.

We included the following predictive factors in the model:

- Age.
- Gender.
- Diabetes.
- Smoking status.
- Obesity.
- Open fractures.
- Peripheral arterial disease.

We collected all data on predictive factors from the clinical information system and using patient interviews.

### Outcome (measures) and follow-up

#### Definition and measurement

We defined time to failure (nonunion, implant failure or loss of reposition) as the primary outcome. We analyzed the following secondary outcomes:

- Physical functioning, as given by three items from the SF-36 questionnaire (difficulty in climbing one stair, difficulty in climbing more stairs, difficulties in stooping; each item offering three answer categories: much difficulties, some difficulties or no difficulties; score 0 to 9).
- Subjective fracture related pain (given on a numeric rating scale (NRS) 0–10).

#### Data collection and follow-up

We collected outcome data with a questionnaire sent via post. In case of no response 3 weeks later, we sent a reminder invitation by SMS and tried to reach the non-responders by phone. In addition, we checked the clinical information system for occurrence of failure treated in our center. We planned to exclude failures because of surgical errors but in none of the implant failures an obvious surgical error was recognized.

We assessed outcomes 6 months after surgery. Last patient out was July the 31st. Cut-off date for data collection was November the 30th. This means the follow-up period was shortened according to the cut-off date for patients who were recruited in the last 2 months. For the last included patient the follow-up was 4 months. We decided to accept a follow-up shorter than 6 months, even though definition of healing is usually referred to 6 months because we analyzed failure with a Cox regression. In this analysis, patients with a shorter follow-up than 6 months are not counted as no failure but the observation time is censored at the study end.

### Sample size

Studies suggest that five outcomes per variable are necessary in regression models of binary data [12]. We included seven predictors in the model. This means, at least 35 events should be observed in total. We anticipated a failure rate of 12 %. Consequently, we planned to include 292 patients in the final analysis.

### Data management and statistical analysis

#### Data management

We entered all data in a standardized case report form. One investigator made all entries and a second investigator verified the entries.

#### Statistical analyses

For the analysis of the primary outcome, we used a multiple stratified (according fracture localization) Cox proportional hazard regression model. We entered all predictive factors simultaneously in the model. It should be noted that we were interested in factors that are potential candidates for a risk prediction model, not in causal relations. We performed a subgroup analysis according to the localization (femur vs. below the knee).

The association between the predictive factors and physical functioning and pain was assessed using analysis of variance (ANOVA). For this analysis, we replaced missing values using multiple imputation (fully conditional Markov Chain Monte Carlo, 10 datasets).

We calculated 95 % confidence intervals (CIs) for all effect estimates.

## Results

### Population

We recruited 204 patients. For 26 (12.7 %) we had no data on failure (2 died, 24 could not be reached e.g. moved and 2 for other reasons). In the analysis of pain and physical functioning, 172 (84 %) patients were included (in addition to the lost patients above, 6 more did not respond any question on patient reported outcomes). For 24 patients the follow-up period was < 6 months. Although, this targeted sample size was not reached, we nearly satisfied the number of observations per variable needed (33 instead of 35) because the failure rate was higher than expected.

Table 1 shows the baseline characteristics.

**Table 1:** Baseline characteristics

Predictive factor	
Age (mean ± SD)	51.39 (± 17.05)
Female	90 (44.1 %)
BMI (mean ± SD)	26.01 (± 5.85)
Obesity (BMI ≥ 30)	37 (18.1 %)
Smoker	75 (36.8 %)
Smoking intensity (package years, mean ± SD)	18.62 (± 17.66)
Smoker type (≥ 10 package years)	47 (23 %)
Diabetes (yes/no)^a^	11 (5.4 %)
Peripheral arterial disease	5 (2.5 %)
Open fracture	29 (14.2 %)
Localization	
* Tibia*	88 (43.1 %)
* Femur*	49 (24.0 %)
* Malleolus*	27 (13.2 %)
* Calcaneus*	17 (8.3 %)
* Fibula*	23 (11.3 %)

*BMI* body mass index, *SD* standard deviation

^a^type I and II not differentiated

### Primary outcome: failure

**Fig. 1:**
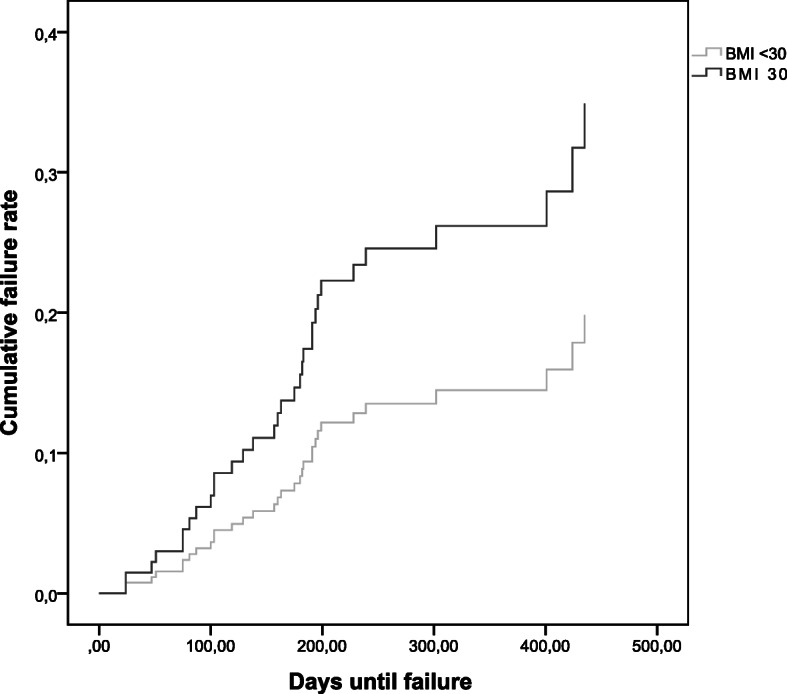
Survival plot for BMI

**Fig. 2:**
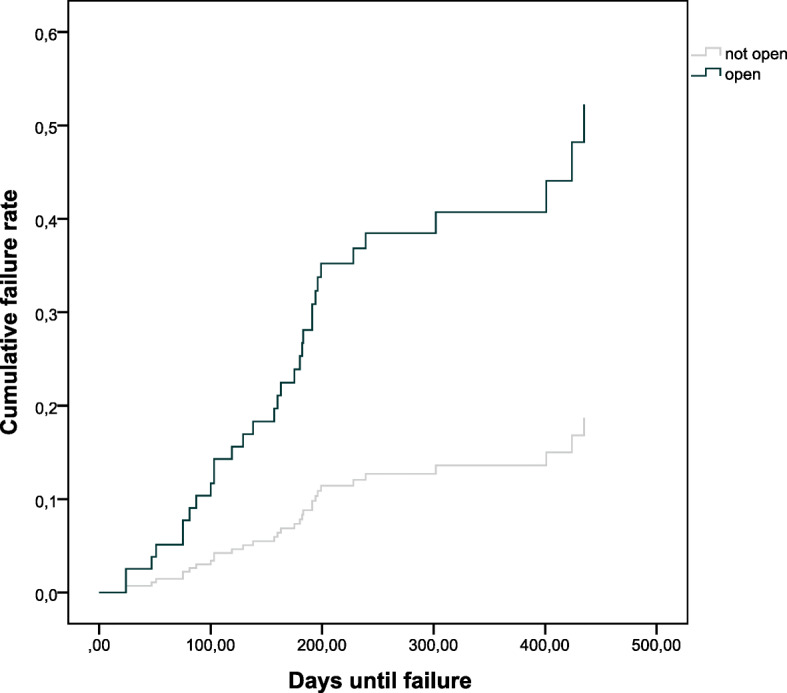
Survival plot for open fracture

Table 2 shows the results of the multiple regression analysis. Figures 1 and 2 show the unadjusted survival curves for BMI and open fracture, respectively.

**Table 2:** Cox regression model of predictive factors for failure

Predictive factor	Hazard Ratio (95 % confidence interval)
Age (≥ 65 vs. < 65)	1.51 (0.63 to 3.60)
Female (yes vs. no)	1.25 (0.55 to 2.81)
BMI (≥ 30 vs. < 30)	2.54 (1.12 to 5.80)
Package years (≥ 10 vs. < 10)	1.19 (0.53 to 2.70)
Open fracture (yes vs. no)	5.36 (2.25 to 12.75)
Diabetes (yes vs. no)	1.07 (0.24 to 4.86)
Peripheral arterial disease (yes vs. no)	2.44 (0.29 to 20.72)

Overall, we observed a failure in 33 patients (16.2 %). Of these, 16 had an implant failure, 8 an additional surgery for reposition and 17 a nonunion. Four of the 33 failure patients received antibiotics for treating a wound infection. Most of the failures (compare Figs. 1 and 2) occurred within the first 3 months. Obesity and open fractures were associated with an increased risk of failure.

Age, female gender and smoking of more than ≥ 10 package years were all associated with an increased risk of failure but statistical uncertainty was high for these predictors. Diabetes was not associated with a higher risk of failure. Explorative subgroup analysis suggested that higher BMI was strongly associated with an increased risk of failure in femur fractures but that the association was uncertain in fractures below the knee (adjusted HR 6.18; 95 %CI 1.42 to 26.95 vs. adjusted HR 0.77 95 %CI 0.24 to 2.44). For the other predictors the association was similar in both localizations (data not shown).

### Secondary outcome: pain

Table 3 shows the results of the ANOVA. After 6 months, 27 % of patients had no pain. Average pain after 6 months was 2.43 (95 %CI 2.08 to 2.79) and the median pain was 2 (inter-quartile-range 2–4). For all predictors, the association with pain was only slight.

**Table 3:** Variables influencing pain

Variable	Regression coefficient (95 % confidence interval)^a^
Intercept	2.89 (1.76 to 4.00)
Age (≥ 65)	-0.04 (-0.92 to 0.84)
Female	-0.02 (-0.71 to 0.68)
BMI (≥ 30)	-0.36 (-0.51 to 1.24)
Package years (≥ 10)	0.06 (-0.72 to 0.82)
Diabetes (yes)	0.79 (-0.70 to 2.28)
Peripheral arterial disease (yes)	0.48 (-1.60 to 2.56)
Open fracture (yes)	0.57 (-0.36 to 1.51)
Localization (femur)	-0.54 (-1.37 to 0.29)

^a^minus indicates a positive impact (reduction in pain)

### Secondary outcome: physical functioning

Results of the multivariable ANOVA for physical functioning are presented in Table 4. No difficulties in any physical task (climbing one stair, climbing more stairs, stooping) after 6 months were reported by 20 % of patients. The median physical functioning score was 6 (lower quartile: 5; upper quartile: 8). Physical functioning was associated with the same factors that increased failure rates, namely BMI and open fracture. In addition, patients with peripheral arterial diseases reported more difficulties with physical functioning.

**Table 4:** Variables influencing physical functioning

Variable	Regression coefficient (95 % confidence interval)^a^
Intercept	6.97 (6.13 to 7.81)
Age (≥ 65)	-0.16 (-0.80 to 0.48)
Female	-0.44 (-0.95 to 0.07)
BMI (≥ 30)	-0.70 (-1.38 to -0.03)
Package years (≥ 10)	-0.18 ( -0.84 to 0.48)
Diabetes (yes)	-0.10 (-1.21 to 1.01)
Peripheral arterial disease (yes)	-1.80 (-3.38 to -0.21)
Open fracture (yes)	-0.80 (-1.54 to -0.07)
Localization (femur)	0.14 (-0.56 to 0.82)

^a^minus indicates a negative impact (more difficulties)

## Discussion

### Key findings

The present study prospectively assessed the association between several predictors and negative outcomes of osteosynthesis over a 6 months period.

We found that open fractures and obesity in femoral fractures were associated with an increased failure risk. This knowledge might increase the clinical information on which the surgeon has to form his personal decision. We found that older age, female gender and arterial peripheral diseases might be associated with a moderately increased failure risk, however statistical uncertainty was high for these variables. Diabetes was associated with a marginal increase of failure risk but the estimate was statistically highly imprecise.

None of the analyzed variables showed a clinical relevant association with long-term pain.

The same variables, which were associate with an increased failure risk, namely obesity and open fracture were also associated with a reduced physical functioning.

### Limitations

The main limitation of our study is the small sample size. Because of this, the effect estimates for age, gender and peripheral arterial disease were uncertain or very uncertain. Another consequence of the small sample size is the statistical need to group the different fracture localizations and types. Our stratified analysis did not suggest strong heterogeneity in the strength of associations as a function of localization. Nevertheless, factors may be ovelooked, which are only important for one or some localizations or fracture types (e.g. fibula).

### Interpretation of results in view of other evidence

Our results are in agreement with previous studies on failure and re-operation. As in our study most of these studies found that BMI (weight and height) and features of the fracture are important predictors for failure of the osteosynthesis [1–4]. Like our study, other studies found high nonunion rates in the lower leg and in obese patients for femoral neck fractures [4]. Femoral neck fractures were a large share in our study as well. Reason for the association of BMI and failure may include technical difficulties to reach the bone and increased soft tissue damage. In addition, worse perfusion might hinder healing and increase risk of infection. Furthermore, we suppose that the BMI might be a surrogate for the mechanical load. The reason that this association with BMI is not evident in fractures below the knee may be that a large share of these fractures were sport or work injuries in non-obese patients.

Until today, the learning process in orthopedic trauma surgery is still primarily based on individual learning from the patients a surgeon has treated (case based learning). Given the currently available opportunities for clinical learning and learning based on usually entity specific scientific publications, the surgeon is caught in a dilemma which is defined by the often limited applicable evidence on the one hand and the cognitive limits of case based learning on the other hand. Information on predictive factors could be one component to support clinical decision making. However, in this and previous studiesthe influence of localization and severity of the fracture was heterogeneous [1, 3, 5, 9, 13]. In particular, the effect size and consequently the clinical importance differed. In the view of the possible variations of fractures, the diversity of classifications systems and their manifold possible resulting classifications (e.g. AO classification), this heterogeneity seems quite impossible to handle using traditional approaches. The findings of this study and previous studies on this topic raise the questions if evidence from “simple” prediction models and standard implementation approaches (e.g. clinical practice guidelines) could support the surgeon’s pre - and intra- operative decision making process significantly and thus could be a valuable approach for getting evidence into trauma care in general. Combining detailed clinical data, data on the surgical technique, imaging data and laboratory data probably would increase the accuracy of prediction. Moreover, building different prediction models for different localizations and fracture types separately could probably increase the performance of a prediction model. However, because of the heterogeneity of fractures and the large amount of possible relevant information, these would require processing big data in clinical routine. One future possibility, which might overcome this challenge might be developing standardized heuristics or algorithms (i.e. artificial intelligence based decision support systems) and implementing these using real-time clinical decision support systems. However, to our knowledge such approaches have received little attention in orthopedic trauma surgery so far [14].

As previously observed, we found no association with pain among the factors considered. This suggests that patient characteristics (e.g. age) or injury related factors (e.g. complexity of fracture) are not the most important determinants but that pre-existing pain, psychological factors and socioeconomic factors might have a stronger association [15, 16]. We neither found clinical important determinants for failure risks and physical functioning with exception to those that generally decrease mobility (obesity and comorbidity) [15]. Thus, it appears that it is not very important to consider the effect of these variables on pain and physical functioning in the decision process on the individually appropriate osteosynthesis.

### Generalizability

On the one hand, we recruited patients only in one urban level I trauma center. This might reduce the generalizability of our results because the patient population might be different in other regions and other centers. Moreover, we cannot exclude that the results may vary between different centers. We believe that center associated variables (e.g. surgical skills) probably have an effect on absolute failure rates but have only little effect on the relative risks for a factor (e.g. the relative risk of open factures across different centers is similar). However, in particular, time to surgery could have an influence on results and might differ in other countries and less urban regions. On the other hand, we applied broad inclusion criteria and our center covers a broad urban as well as rural catchment area. Therefore, it can be assumed that our patient population is quite representative for the “general” lower limb fracture population regarding cause of accident, patient characteristics and fracture characteristics [4, 17].

## Conclusions

In this prospective cohort study, we found factors preoperatively measurable, which appear promising for predicting the failure risk of an internal osteosynthesis in a traumatic lower limb fracture. In particular, obesity and open fractures are associated with the increase of risk of failure. In addition, older age, female gender and peripheral arterial disease tend to be associated to the increase of failure rate. Our results suggest that the combination of a few patient characteristics (e.g. age, BMI, morbidity), localization of the fracture and severity of the fracture might be candidates to predict failure, because they are predictive across different types of fractures at the lower limbs. However, considering the diversity of fractures and fracture classifications, a formalized computer based risk classification could probably increase reliability and feasibility of using fracture related information for predicting failure. A mobile device based risk prediction model combining patient characteristics and computer processed fracture information (e.g. x-ray data), might enable the estimation of the precise individual risk at bedside and therefore could be a convenient tool for routine use to support clinical decision making.

## Data Availability

The datasets used and/or analyzed during the current study available from the corresponding author on reasonable request.

## Abbreviation

BMI: Body-Mass-Index
